# Adjuvant Therapy With Mushroom Polysaccharides for Diabetic Complications

**DOI:** 10.3389/fphar.2020.00168

**Published:** 2020-02-28

**Authors:** Xue Jiang, Weiqi Meng, Lanzhou Li, Zhaoli Meng, Di Wang

**Affiliations:** ^1^ Department of Translational Medicine Research Institute, First Hospital, Jilin University, Changchun, China; ^2^ School of Life Sciences, Jilin University, Changchun, China

**Keywords:** polysaccharides, structure, mushroom, diabetes, diabetic complications

## Abstract

**Background:**

Diabetic complications seriously endanger the health of most diabetic patients around the world. Most chemical hypoglycemic agents have adverse effects and are unable to improve the progression of diabetic complications. In recent years, a number of medicinal herbs have become increasingly popular for the treatment of diabetic complications due to their relative safety. Polysaccharides extracted from medicinal herbs with multiple pharmacological activities and low toxicity have been reported to be useful in the treatment of diabetic complications.

**Methods:**

Primary studies with keywords including polysaccharide and diabetic complications were retrieved from the Web of Science and NCBI databases and were read and analyzed.

**Results:**

Mushroom polysaccharides were proven to have positive effects on diabetic complications.

**Conclusions:**

We studied the effects of mushroom polysaccharides on hyperglycemia and as adjuvant therapies for diabetic complications and summarized the applications and limitations of mushroom polysaccharides to better understand their application for the treatment of diabetic complications.

## Introduction

Diabetes mellitus (DM) is a type of chronic metabolic and noncommunicable disease, DM and its complications affect humans worldwide and result from unhealthy lifestyle, lack of exercise, overweight, excessive intake of high sugar food, and genetic and many other factors (Zhou et al., 2014; Unnikrishnan et al., 2016; Bailey et al., 2017). According to statistics from the International Diabetes Federation, in 2015, there were 415 million people suffering from diabetes globally (Zimmet et al., 2016). It is predicted that 592 million patients suffering from DM will die due to various complications by 2035 (Tao et al., 2015).

DM is a comprehensive disease in which patients have long-term metabolic abnormalities due to chronic hyperglycemia (Alam et al., 2014; Shi et al., 2018), and the main symptoms include polyuria, weight loss and poor vision (American Diabetes A, 2014). According to the pathogenesis, diabetes can be classified as follows: (1) type 1 diabetes mellitus (T1DM), which is caused by β-cell destruction and usually leads to absolute insulin deficiency; (2) type 2 diabetes mellitus (T2DM), which predominantly shows insulin resistance (IR) with relative insulin deficiency; (3) gestational diabetes mellitus (GDM), which is defined as diabetes diagnosed during pregnancy; And (4) specific types of diabetes caused by other factors, such as monogenic diabetes mellitus (including neonatal diabetes and maturity-onset diabetes of the young (MODY)), diseases of the exocrine pancreas (such as fibrocalculous pancreatic diabetes mellitus (FCPD) and pancreatitis) and drug/chemical-induced diabetes (Coustan, 2013; American Diabetes A, 2014; Unnikrishnan et al., 2016; American Diabetes A, 2018). Hyperglycemia is a hallmark of all forms of diabetes, and hypertension and lipoprotein metabolism abnormalities can also be observed in diabetic patients (Gavin et al., 2000; Giacco and Brownlee, 2010).

A series of complications caused by diabetes may aggravate pain in patients and even cause death. Diabetic complications include macrovascular and microvascular diseases (American Diabetes A, 2013; Zhou et al., 2014). In addition, compared with healthy people, patients with diabetes have fewer collateral vessels, which may increase the rate of lower limb amputation and mortality of ischemic events (Giacco and Brownlee, 2010). Complications such as cardiovascular disease, kidney disease, neuropathy, blindness, and amputation may take be responsible for the increased morbidity, disability, and mortality among people with diabetes (Deshpande et al., 2008; Papatheodorou et al., 2016). Therefore, to alleviate the suffering of patients and avoid serious outcomes, diabetic complications need to be diagnosed and treated in a timely manner.

Some medications for diabetes therapy, including sulfonylureas, α-glucosidase inhibitors, thiazolidinediones, meglitinides, and biguanides have been used for many years. New drugs with new targets, such as SGLT-2 inhibitors and DPP-4 inhibitors, have been developed in recent years. The side effects of these drugs seriously endanger the health of patients and are difficult to address, as summarized and shown in **Table S1**. Adverse reactions, undesirable side effects and the huge cost of synthetic drugs are some of the reasons for the trend of searching for alternative natural agents with low price and side effects for the treatment of DM and especially its complications (Mohammed et al., 2007; Vitak et al., 2017).

Polysaccharides extracted from mushrooms have multiple pharmacological activities and low toxicity and have attracted more attention from researchers (Ganesan and Xu, 2019); assessing polysaccharides with therapeutic effects on diabetic complications has become an important research area (Wang D. et al., 2019). Mycelium zinc polysaccharides from *Pleurotus djamor* show hepatoprotective and renoprotective effects through the regulation of oxidative stress (OS) and exhibit potential effects on diabetic complications (Zhang et al., 2015). Polysaccharides isolated from *Ganoderma lucidum* attenuate myocardial collagen cross-linking by augmenting antioxidant enzyme activities and decreasing advanced glycation end product (AGE) levels in diabetic rats and may thus have the potential to treat myocardial fibrosis (Meng et al., 2011). Yunzhi polysaccharides show protective effects on bone properties in a DM-induced bone-loss model by improving hyperglycemia (Chen et al., 2015). Therefore, finding effective mushroom polysaccharide agents to treat diabetes and its complications is a promising direction.

In this review, we summarized the molecular pathogenesis of diabetic complications and the therapeutic role of mushroom polysaccharides and discussed the potential possibility of mushroom polysaccharides as useful drugs in preventing diabetic complications. We hope our discussion provides evidence for the use of mushroom polysaccharides as nutraceuticals or commercial drugs in diabetic complications.

## Pathogenesis of Therapeutic Strategies for Diabetic Complications

**Figure 1:**
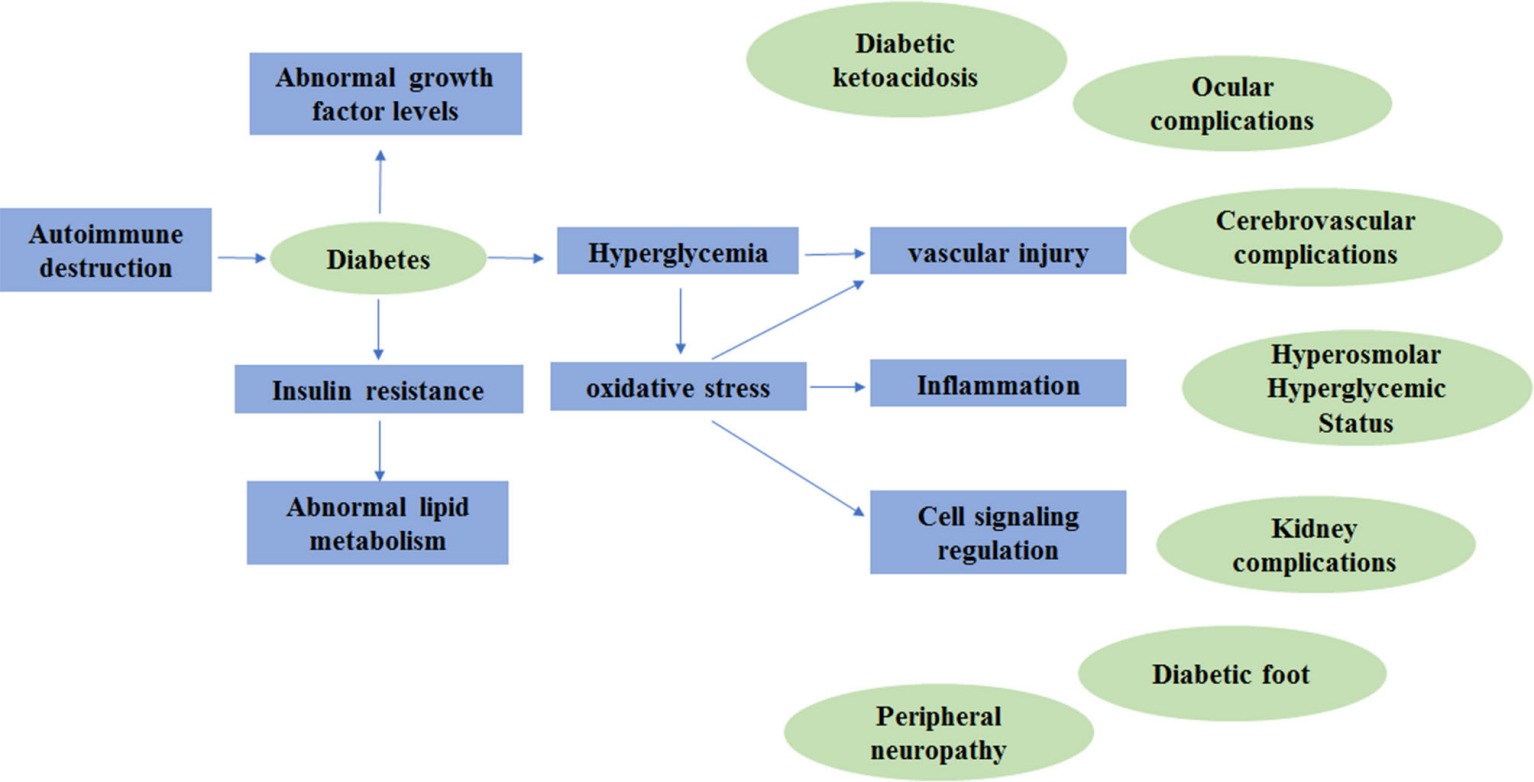
The molecular pathogenesis of diabetes mellitus and its complications.

DM does not cause the disability or death of patients directly; however, its complications due to the metabolic abnormalities including diabetic nephropathy (DN), diabetic retinopathy (DR) and peripheral neuropathy may increase morbidity, disability and mortality (Giacco and Brownlee, 2010; Papatheodorou et al., 2016). Diabetes complications can be divided into acute complications and chronic complications (Greene, 1986). The acute complications of diabetes are severe and rapidly developing, posing a serious threat to patients' lives (Barrett et al., 2017). Fortunately, death caused by acute complications is almost completely preventable (Klafke et al., 2015). The chronic complications of diabetes are chronic, occult, lifelong and progressive diseases that can cause lesions in multiple systems throughout the body (Pinhas-Hamiel and Zeitler, 2007). Here, we summarize the molecular pathogenesis of DM and its complications, which are shown in **Figure 1**.

### Acute Complications

#### Diabetic Ketoacidosis

Diabetic ketoacidosis (DKA) is a life-threatening and serious complication of diabetes (American Diabetes A, 2014). DKA is more common in young people suffering from T1DM and is characterized by a lack of insulin, severely high levels of blood glucose, metabolic acidosis and ketosis (Fayfman et al., 2017; Modi et al., 2017). Continuous insulin injection and repeated low-dose injections of insulin are common treatments for DKA and are simple, safe and effective (Dhatariya and Vellanki, 2017). However, due to the involvement of medical facilities, insulin treatment has a high cost (Kreider, 2018).

#### Hyperosmolar Hyperglycemic Status

Hyperosmolar hyperglycemic status (HHS), which is characterized by high levels of blood sugar and insulin deficiency, is one of the most serious complications of diabetes and is commonly observed in adult and elderly patients with T2DM (American Diabetes A, 2014; Chang et al., 2016). Urinary tract infection and pneumonia are two common causes of HSS. HHS can coexist with DKA. HHS has a lower hospitalization rate and higher mortality rate than DKA (Fayfman et al., 2017). Current treatment options are fluid and electrolyte infusion or insulin infusion; certain minerals, such as sodium and potassium, can be quickly replaced during treatment; however, the treatment process is complicated and may cause electrolyte imbalance (Bhowmick et al., 2005; Fayfman et al., 2017; Baldrighi et al., 2018). Therefore, early diagnosis and management are vital in HHS treatment.

#### Lactic Acidosis

Abnormal glucose metabolism in diabetic patients causes elevated levels of lactic acid and leads to acidosis (Reddy et al., 2015). The main causes of lactic acidosis are hypoxia, congenital metabolic abnormalities and the use of drugs such as metformin (Reddy et al., 2015; Seheult et al., 2017). Patients with lactic acidosis often have additional symptoms, including nausea, abdominal pain and lethargy. It has been noted that metformin is one of the best options for the treatment of T2DM; however, metformin treatment for diabetes may sometimes lead to lactic acidosis (Inzucchi et al., 2014; Lalau et al., 2017).

### Chronic Complications

#### Diabetic Nephropathy

DN, as a chronic complication of diabetes, is a main cause of end-stage kidney disease (Flyvbjerg, 2017; Zhang J. et al., 2018), which occurs in patients with DM, and reduced kidney function involved in hyperglycemia-induced renal hyper filtration and injury, AGE-induced increased OS, activated protein kinase C (PKC)-induced increased production of cytokines, chemokines, and different inflammatory and apoptotic signals (Bhattacharjee et al., 2016). Detachment of podocytes from the glomerular basement membrane is also considered a key factor in the development of DN (Sawada et al., 2016). Chronic inflammation and the production of glomerular and tubular hypertrophy caused by OS are early features of DN (Miranda-Diaz et al., 2016). Currently, early prevention, glycemic control, blood pressure control, and the inhibition of the renin-angiotensin system (RAS) are four major treatment paradigms (Gallagher and Suckling, 2016). RAS-blocking medications are the best evidence-based treatments for DN; however, they cannot prevent end-stage renal disease and may lead to adverse reactions, such as acute kidney injury (Umanath and Lewis, 2018). In recent years, a growing body of research has shown the effective roles of mushroom polysaccharides in DN. Polysaccharides isolated from *Auricularia auricula* significantly promote glucose metabolism and reduced blood glucose levels and protect against DN by regulating blood urea nitrogen, creatinine, urine protein and inflammation-related factors (Hu et al., 2017). Residue polysaccharides from *Flammulina velutipes*, which can be used to treat DN, protect against OS by removing reactive oxygen species (ROS) and lowering the content of malondialdehyde (MDA) in the kidney (Lin et al., 2016). Available data also suggest that proteoglycans extracted from the fruit body of *G. lucidum* protects kidney function through antioxidant activities (Pan et al., 2014).

#### Diabetic Ocular Complications

Eye diseases caused by diabetes include DR, cataract and macular degeneration (Geloneck et al., 2015). The longer the duration of diabetes, the more complications occur, and the higher the risk of visual impairment or even blindness (Ahmed et al., 2017).

DR, which is arteriosclerotic plate deposition caused by hyperglycemia, causes lesions of capillaries, arterioles and venules in the retina (Scanlon, 2019), subsequently causing retinal vascular inflammation (Calderon et al., 2017). DR is considered the most common microvascular complication leading to blindness (Dehdashtian et al., 2018). Approximately 33.33% of diabetes patients suffer from DR (Jenkins et al., 2015). The development of DR is also associated with high blood glucose, high blood pressure, abnormal lipid profiles, gravidity, sexual maturity and cataract surgery (Dehdashtian et al., 2018; Mariam et al., 2018). In addition, aging, genetic susceptibility and ethnicity may also influence the development of DR in DM (Scanlon, 2019).

DM can affect ocular structures, and cataract is the most common ocular complication (Kelkar et al., 2018). Cataract symptoms mature earlier in DM patients than in other populations (Peterson et al., 2018). Different types of mechanisms have been proposed for the pathogenesis of cataract caused by DM, such as the polyol pathway, osmotic stress, OS and autoimmunity (Kiziltoprak et al., 2019). Cataract surgery is a common therapeutic strategy; however, unsuccessful surgery increases intraocular inflammatory cytokines (Kelkar et al., 2018).

The macula is located in the center of the retina and is responsible for central vision. When a fragile blood vessel ruptures, the recumbent fluid in the retina becomes thickened, causing central vision loss; this is called diabetic macular edema (DME) (Simo et al., 2006; Vieira-Potter et al., 2016) and is commonly observed in T2DM (Tong et al., 2001). The existing treatment strategies for DME are macular laser photocoagulation and intravitreal injection (Moisseiev and Loewenstein, 2017).

#### Diabetic Foot

Diabetic foot (DF) includes diabetes-related foot ulcers (DRFUs) and Charcot arthropathy, both of which are the result of a combination of neurological trauma and vascular disease (DiPreta, 2014). DF is difficult to treat and easy to relapse, which places a heavy burden on individuals, families and society (Lim et al., 2017; Paisey et al., 2018).

DRFUs are ischemic, neurological and ischemic lesions of the foot that can cause different degrees of infection, ulcers and risk of gangrene and amputation (Ahmad, 2016; Fernando et al., 2017). The probability that a diabetic patient will develop DRFUs in their lifetime is 25% (Singh et al., 2005). Progressive atrophy of skin connective tissue, OS and accumulation of AGEs are the main mechanisms that affect DRFUs (Wear-Maggitti et al., 2004; Ahmad, 2016). Debridement of DRFUs is an essential approach (Bergin et al., 2012). In Australia, amputation is a common phenomenon because of DRFUs (Chuter et al., 2019).

According to previous conclusions, the pathogenesis of Charcot arthropathy is as follows. Diabetic neuropathy and vascular disease affect the musculoskeletal system and lead to the deterioration of inflammation, which can cause the destruction or deformity of bones and joints (Caldino-Lozada et al., 2017). It usually occurs in the feet and ankles of people with diabetes. Adequate control of diabetic hyperglycemia is a prerequisite for treatment, and conservative treatment is the general method. Additionally, the relief of foot pressure and bed rest are basic treatments (Stanley and Collier, 2008).

#### Diabetic Heart Disease

Diabetes can lead to a range of cardiovascular diseases, including ischemic heart disease and heart failure (Dutta et al., 2001; Kovacic et al., 2014). Deaths due to cardiovascular disease account for 80% of total deaths from T2DM (Yandrapalli et al., 2017). Surgery is the preferred treatment for diabetic heart disease, and during the operation, blood glucose and blood pressure should be strictly monitored (Benedetto et al., 2016). For oral medicine, the benefits of aspirin for secondary prevention of DM are currently undisputed (Capodanno and Angiolillo, 2016). However, the risks of myocardial infarction, coronary heart disease and cardiovascular death are not significantly reduced with aspirin treatment (Kunutsor et al., 2017).

#### Diabetic Cerebrovascular Disease

Diabetic cerebrovascular disease, a main cause of death in DM patients, is caused by disorder of substance metabolism, leading to intracranial large and small vessel diseases. Approximately 20–40% of patients with T2DM suffer from cerebral blood vessel diseases (Bal et al., 2014; Zhou et al., 2014; Barrett et al., 2017). Hyperglycemia causes OS and induces vascular damage; at the same time, hyperglycemia results in the consumption of endothelial cell NO and reduces the effectiveness of vasodilation (Bailey et al., 2017). Hyperglycemia can also cause vasogenic edema, increase thrombosis, reduce cerebral blood flow and damage automatic adjustment of the brain (McCormick et al., 2010). Cerebrovascular disease can manifest as three types of disorders (Zhou et al., 2014). (1) One of these disorders is silent stroke (SS). The risk of ischemic stroke increases continuously with the duration of DM (Banerjee et al., 2012). (2) The second type of disorder is cerebral small vessel disease (SVD) and acute cerebrovascular disease. SVD has been shown to be associated not only with the incidence of stroke but also with diabetic microangiopathy (Umemura et al., 2017). Available data also suggest that controlling blood glucose is one of the strategies to treat acute cerebrovascular disease (Mo et al., 2019). (3) The third type of disorder is cerebral arteritis. It is the main mechanism of T2DM and essentially an inflammatory reaction (Ross, 1999). Early diagnosis and intensive glycemic control can reduce diabetic microvascular complications and possibly macrovascular complications (Khunti and Seidu, 2019).

#### Diabetic Peripheral Neuropathy

Another common complication is diabetic peripheral neuropathy (DPN), which is the most troublesome for patients. Thirty percent of T2DM patients will develop DPN, and 50% of adults with DM will be affected by DPN (Bailey et al., 2017; Yorek, 2018). Diabetic hyperglycemia may drive the progression of DPN (Singleton et al., 2001). Nerve cells are vulnerable targets of diabetes, excessive glucose can hinder adenosine triphosphate (ATP) production and ROS overproduction, and nerve cells are impaired by OS (Giacco and Brownlee, 2010; Bailey et al., 2017). The symptoms are pain, numbness, paresthesia and ulceration in the limbs (Yorek, 2018). Unfortunately, early DPN is not easily diagnosed; therefore, DPN increases the risk of patient infection and amputation (Azhary et al., 2010; Wang et al., 2017). Treatment for DPN remains difficult and focuses on improving blood circulation or ameliorating OS (Vallianou et al., 2009; Sandireddy et al., 2014). Acupuncture may ameliorate DPN symptoms effectively by improving blood circulation in these patients (Hsiao and Tsai, 2008). Medications for DPN are usually analgesic anti-inflammatory drugs; however, they can only alleviate pain symptoms and cannot address the potential mechanism of DNP (Javed et al., 2015).

### Mechanism of Diabetic Complications

The onset of diabetes is due to autoimmune destruction of pancreatic β cells, causing insulin deficiency and abnormalities in insulin-related metabolic processes. Insulin deficiency leads to abnormal carbohydrate, fat and protein metabolism in diabetes (American Diabetes A, 2014). The mechanisms of diabetic complications vary, as explained above, but there are some major mechanisms, such as the production of AGEs, the formation of a proinflammatory microenvironment and the induction of OS (Papatheodorou et al., 2016).

Abnormal glucose metabolism in diabetic patients leads to overproduction of superoxide, which causes diabetes complications mainly through five processes: (1) polyol pathway flux, (2) increased AGE formation, (3) increased expression of AGEs and its activating ligand receptor, (4) activation of PKC, and (5) overactivity of the hexosamine pathway (Singh et al., 2005). There is currently a hypothesis that the above five processes are all activated by “overproduction of the mitochondrial ROS” (Brownlee, 2005). AGEs are the main pathogenic products of glucose metabolism disorder and can induce OS in multiple pathways and at multiple sites. Increased levels of AGEs in hyperglycemia can induce vascular inflammation (Pantham et al., 2015). AGEs can indirectly activate the AGEs-RAGE signaling pathway and increase low levels of intracellular ROS. ROS can participate in cell signaling regulation through their oxidation. Overaction of the AGEs-RAGE signaling pathway mediates the inflammatory response, autophagy, and apoptosis through OS, resulting in vascular and neurological damage and leading to diabetic complications (Hussain et al., 2013).

IR significantly blocks the suppressive effects of insulin on fatty acid oxidation and increases free fatty acid (FAA) release from adipose tissues. The lack of insulin-stimulated malonyl-CoA production leads to increased oxidation of FFA in endothelial cells (Moule and Denton, 1997; Giacco and Brownlee, 2010). Hyperglycemia causes excessive production of mitochondrial electron transport chain superoxide in endothelial cells, and the oxidation of FFA provides the same electron donors; hence, FFA leads to the increased production of mitochondrial electron transport chain superoxide (Du et al., 2006). It can activate multiple inflammatory signals, inactivate two important anti-atherosclerotic enzymes and activate diabetic vascular complications caused by microvascular injury (Brownlee, 2001).

Cells that are directly sensitive to hyperglycemia damage in diabetic patients, such as endothelial cells, cannot reduce glucose uptake or change the glucose transport rate, which make themselves the exposure to abnormally high glucose conditions and lead to diabetic complications associated with vascular injury (Giacco and Brownlee, 2010).

Growth factors are broadly defined as active polypeptides with bioactivity (Zarei et al., 2018). As the research progressed, researchers have begun to believe that abnormal expression of growth factors plays a role in the development of diabetic complications (Shi et al., 2018). TGF-β1, a fibrotic cytokine, can cause renal cell hypertrophy, regulate the generation of extracellular matrix molecules and induce the production of chemokines in the proximal tubules of the kidney, which promotes DN to some extent (Qiao et al., 2017). Experiments have shown that streptozotocin (STZ) triggers DR by increasing the levels of TNF-α, IL-1β, and IL-6 (Gao et al., 2017). An increasing number of studies have suggested that growth factors are involved in the pathogenesis of diabetic complications; however, the direct causal relationship between growth factors and complications has not been clearly demonstrated because of the complex process of disease formation and the intricate relationships between growth factors (Shi et al., 2018).

## Structure-Activity Relationship of Mushroom Polysaccharides

Mushroom polysaccharides consist of glucans. D-glucose monomers make up glucans, two glucose units combined together *via* the α-glycosidic bond (carbon in an axial position) or β-glycosidic bond (carbon in an equatorial position), including C1 and C6, C1 and C4 or C1 and C3. The linkages of α (1→3) or β (1→3) or (1→6) can form heteroglycans, including mannose, galactose, arabinose, fucose and xylose. They combine with protein residues to form polysaccharide-protein complexes. Polysaccharides in a certain of mushroom species combine with peptides/proteins to form polysaccharide-protein/peptide complex groups (Pandya et al., 2019). Polysaccharides are difficult to study because they are very complicated and not encoded in the genome. Polysaccharides were confirmed to exhibit multiple biological functions, including antidiabetic, antioxidant, immunomodulatory, and hepatoprotective functions in recent decades (Wu et al., 2016). Here, we describe some of the compounds produced by mushrooms. The primary structure of Lentinan (400-800 kDa) contains five (1→3)-β-glucose residues in a linear linkage and two (1→6)-β-glucopyranoside side chains (Zhang et al., 2001). Lentinan can protect against pancreatic β-cell apoptosis and dysfunction by inhibiting ROS generation and exhibits anti-apoptotic effects by preventing JNK and p38 MAPK signaling or/and antidysfunction effects by preventing NF-κB activation (Zhang et al., 2016). Krestin (94 kDa), also known as *Coriolus versicolor* polysaccharide, is a β-glucan-protein complex made up of approximate 25-38% protein residues. The primary constituent monosaccharide is glucose with a handful of other sugar residues like xylose, galactose and mannose (Maehara et al., 2012). Krestin has been reported to ameliorate insulin resistance and hyperlipidemia *via* the regulation of inflammatory cytokine expression (Xu et al., 2016). GFP-N, isolated and purified from the *Grifola frondosa*, is mainly composed of three residues including →2,6)-α-D-Manp-(1 → 4, →3,6)-β-D-Glcp-(1 → and α-L-Araf-C1→ . GFP-N can ameliorate hyperglycemia and protect against kidney and liver injury, and particularly improved hepatic IR *via* the regulation on JNK and IRS1/PI3K signaling (Chen et al., 2019). AAMP is a polysaccharide-enriched fraction isolated from the fruit body of *Amillariella mellea*. It is composed of 58.6% glucose, 19.8% galactose, 18.1% mannose, 3.3% glucuronic acid, and 1.5% fucose and consists of two fractions with molecular weights of 23.3 kDa (the major fraction) and 321 kDa. AAMP exerts hypoglycemic effects and improves IR, likely by decreasing lipogenesis and increasing lipolysis (Yang et al., 2019). IOEP1 (20 kDa, mannose and galactose) and IOEP2 (200 kDa, arabinose) are two *Inonotus obliquus* crude polysaccharides that are pyran-type polysaccharides with glycosidic bonds that are all α- and β-type. They improve glucose consumption and insulin resistance in HepG2 cells and exhibit hypoglycemic effects (Xue et al., 2018). Suilu.A (6383 Da), Suilu.C (8172 Da) and Suilu.S (10710  Da) are three polysaccharides extracted from *Suillellus luridus*; they consist of glucose, mannose, arabinose, xylose and galactose and have been reported to exhibit antihyperglycemic and antihyperlipidemic activities by reducing blood glucose and serum lipid levels, regulating antioxidant status, and reducing liver and kidney damage (Zhang L. et al., 2018). *Pleurotus eryngii* mycelium polysaccharides are furanose-linked through β-type glycosidic bonds, have antioxidant capacity *in vitro* and *in vivo* and improve glucose metabolism and lipid metabolism in diabetic mice (Wang Y. et al., 2019).

## Effects of Mushroom Polysaccharides on Hyperglycemia and Adjuvant Therapy for Diabetic Complications

**Figure 2:**
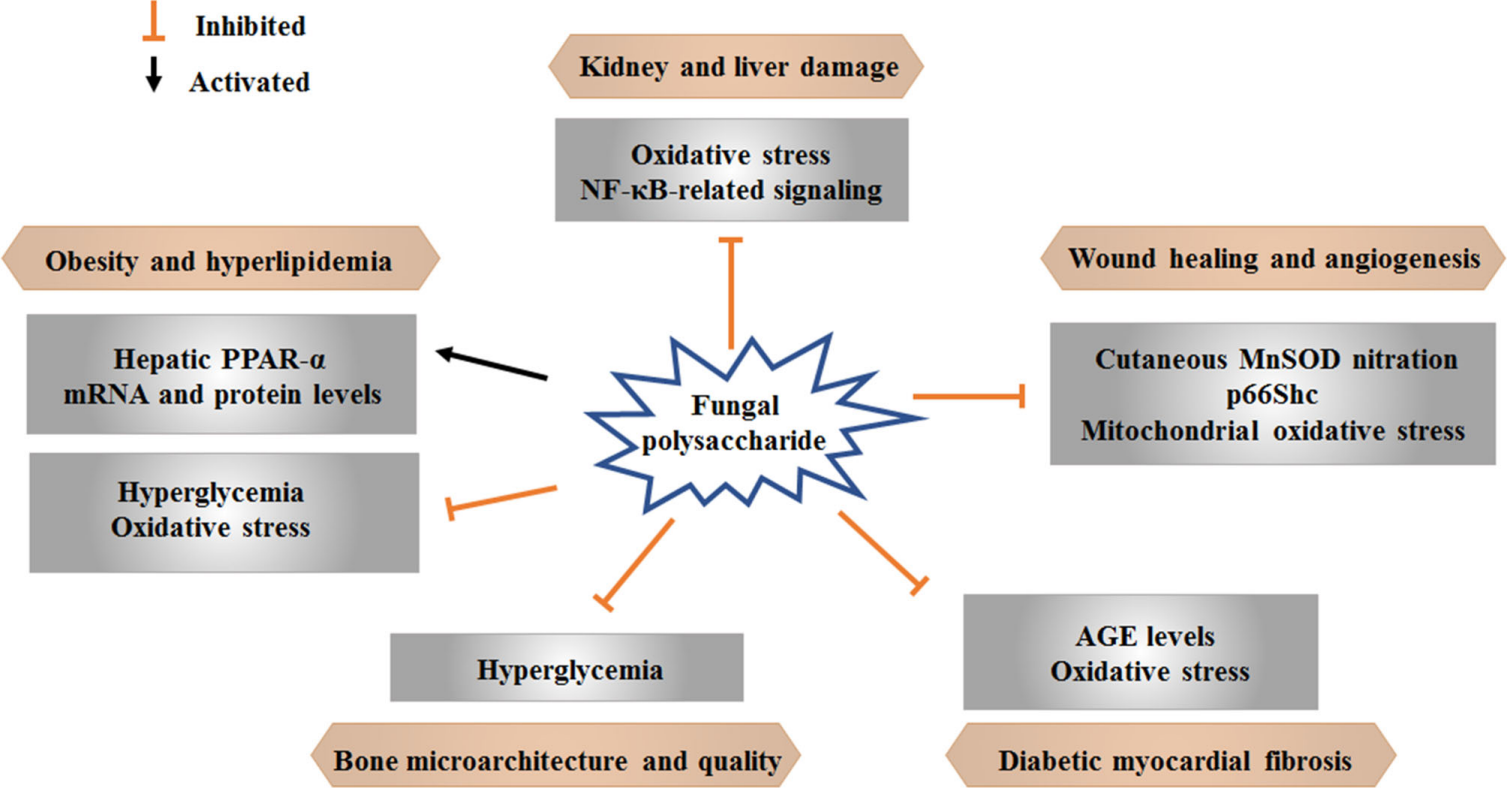
Effects of mushroom polysaccharide on diabetic complications.

Some advanced research on natural products has shown positive effects of therapies for diabetes and related complications. Current findings regarding the effects of mushroom polysaccharides on hyperglycemia and as adjuvant therapies for diabetic complications are summarized here (**Table 1** and **Figure 2**). Studies on mushroom polysaccharides have mainly focused on lipid metabolism, diabetic renal complications, myocardial fibrosis, wound angiogenesis and bone loss; however, almost none of these studies have concentrated on other complications, such as diabetic foot, retinopathy and complications of the nervous system.

**Table 1:** Effects of mushroom polysaccharides on hyperglycemia and as adjuvant therapies for diabetic complications.

Latin name	Polysaccharide	Models	Dose and period	Diabetic and related Complications	Possible mechanisms	Year/References
*Auricularia auricula*	AAP; crude polysaccharide	Intraperitoneal injection of 30 mg/kg STZ in male SD rats for 3 days	100 and 400 mg/kg for 4 weeks	Antidiabetic and antinephritic effects	Modulation of the antioxidative system and NF-κB-related signaling	2017 (Hu et al., 2017)
*Flammulina velutipes*	Ac-RPS, Al-RPS and En-RPS; monosaccharide composition analysis	Three successive intraperitoneal injections of 80 mg/kg STZ in male Kunming mice	200, 400 and 800 mg/kg for 15 days	Antioxidant abilities and protective effects against kidney damage	Serum CRE, BUN, ALB, GLU and renal MDA levels (↓); renal SOD, CAT and GSH-Px levels (↑); alleviation of kidney damage	2016 (Lin et al., 2016)
*Pleurotus djamor*	Ac-MZPS, Al-MZPS and En-MZPS; acidic-, alkalic- and enzymatic-extractable mycelium zinc polysaccharides	Intraperitoneal injection of 120 mg/kg STZ in Kunming mice for 5 days	200, 400 and 800 mg/kg for 2 weeks	Antioxidant abilities and protective effects against kidney and liver damage	SOD, GSH-Px and CAT levels (↑); MDA, ALT, AST, BUN, CRE, TC, LDL-C and HDL-C levels (↓); alleviation of liver and kidney injury	2015 (Zhang et al., 2015)
*Trametes versicolor* (L.:Fr.) Pilát (also known as Yunzhi)	TVP LH-1ePSP; monosaccharide composition analysis	Injection of 65 mg/kg STZ *via* the tail vein in male Wistar rats for 5 days	100 mg/kg for 28 days	Beneficial effects on bone microarchitecture and bone quality in a DM-induced bone-loss model	Protection of bone properties in part through improvement of hyperglycemic control	2015 (Chen et al., 2015)
*Pleurotus tuber-regium*	HP, MP and LP; extracellular polysaccharides	Intraperitoneal injection of 10 mg/kg STZ along with 30 mg/kg nicotinamide every other day for 6 weeks	20 mg/kg for 8 weeks	Attenuation of obesity/diabetes-induced adverse effects through the maintenance of stable fatty acid composition and reversal of obesity and hyperlipidemia	Support of upegulated hepatic PPAR-α mRNA and protein levels and antihyperglycemic properties	2014 (Huang et al., 2014)
*Ganoderma lucidum*	G1-PS; crude polysaccharide	Single injection of 65 mg/kg STZ in SD rats	20 mg/kg for 8 weeks	Hypoglycemic effect	Prevention of apoptosis of pancreatic β-cells and enhancement of β-cell regeneration	2012 (Zheng et al., 2012)
	G1-PS; crude polysaccharide	Intraperitoneal injection of 60 mg/kg STZ in male C57BL/6 mice for 5 days	10 mg/kg, 50 mg/kg and 250 mg/kg	Rescue of delayed wound healing and improvement of wound angiogenesis	Suppression of cutaneous MnSOD nitration, p66Shc and mitochondrial oxidative stress	2012 (Tie et al., 2012)
	GLP-1 and GLP-II; polysaccharide purity: 74.03% and 75.61%	Intraperitoneal Injection of 30 mg/kg STZ in SD rats for 3 days	200, 400 and 800 mg/kg for 16 weeks	Diabetic myocardial fibrosis	Attenuation of myocardial collagen crosslinking related to the decreased level of AGE and augmented activities of antioxidant enzymes	2011 (Meng et al., 2011)
	GL-PS; crude polysaccharide	Intraperitoneal injection of 100 mg/kg STZ in C57BL/6J mice for 2 days	125 and 250 mg/kg for 8 weeks	Improvement of diabetic renal complications	Amelioration of metabolic disorders, oxidative stress and renal dysfunction associated with renal lesions	2006 (He et al., 2006)
	GLPS; crude polysaccharide	Intraperitoneal injection of 35 mg/kg STZ in SD mice for 3 days	200, 400 and 800 mg/kg for 8 weeks	Diabetic myocardial fibrosis	Regulation of oxidative stress and reductions in the levels of AGEs and myocardial fibrosis	2011 (Li, 2011)
		Intraperitoneal injection of 30 mg/kg STZ in SD mice for a week	300 and 600 mg/kg for 12 weeks	Diabetic myocardial fibrosis	Strengthening of antioxidant enzyme activities and downregulated of myocardial CTGF	2014 (Qiao et al., 2014)
*Phallus impudicus*	PIP; Mycelium polysaccharide	Full-thickness skin flap excision model in Wistar rats	Ointment containing 10% *Phallus impudicus* polysaccharides	Wound healing properties	Enhancement of healing with regards to epithelialization, contraction and growth of granulation tissue	2019 (Vyacheslav et al., 2019)
*Tremella aurantialba*	TMP; crude polysaccharide	Intraperitoneal injection of 180 mg/kg alloxan in Wistar rats for 5 days	100 mg/kg for 28 days	Abnormal lipid metabolism and elevated oxidative stress	Regulation of lipid metabolism and decreased oxidative stress	2009 (Zhang et al., 2009)

Obesity and abnormal lipid metabolism are common complications of DM. Extracellular polysaccharides from *Pleurotus tuber* maintain a stable fatty acid composition and regulate hyperlipidemia and obesity by upregulating the levels of hepatic PPAR-α mRNA and protein and exerting antihyperglycemic properties (Huang et al., 2014). *Tremella aurantialba* mycelia polysaccharides regulate lipid metabolism and decrease OS in alloxan-induced diabetic rats (Zhang et al., 2009).

DN is a chronic complication of diabetes and a main cause of end-stage kidney disease. Polysaccharides isolated from *A. auricula* exhibit anti-nephritic properties by modulating OS and the NF-κB pathway in diet/STZ-induced diabetic rats (Hu et al., 2017). Ac-RPS, Al-RPS, and En-RPS are three *F. velutipes* polysaccharides extracted with HCl, NaOH and snailase solution, respectively. Ac-RPS contains Rib (11.3%), Ara (17.7%), Xyl (14.1%), Gal (39.6%), and Glu (17.3%). Al-RPS contains Rib (14.3%), Ara (36.8%), Xyl (12.9%), Gal (22.4%), and Glu (13.6%). En-RPS contains Ara (8.1%), Xyl (9.7%), Gal (71.7%), and Glu (10.5%). The three polysaccharides, particularly 800 mg/kg En-RPS, exhibits renoprotective effects *via* antioxidation (Lin et al., 2016). Three *P. djamor* mycelium zinc polysaccharides (MZPS), including acidic-MZPS, alkalic-MZPS and enzymatic-MZPS, show protective effects on the kidney and liver in STZ-induced diabetic mice by reducing OS (Zhang et al., 2015). *G. lucidum* polysaccharides delay the progression of DN by ameliorating metabolic disorders, OS and renal lesions (He et al., 2006).

Myocardial fibrosis caused by long-term DM may lead to myocardial stiffness development, which is a dangerous element of cardiovascular disease (Asbun and Villarreal, 2006). *G. lucidum* polysaccharides weaken myocardial collagen crosslinking by decreasing AGE levels and increasing antioxidant enzyme activities in diabetic rats and thus is a potential therapy for myocardial fibrosis (Meng et al., 2011). Another study indicated that *G. lucidum* polysaccharide can improve the activities of CAT and GSH-Px, enhance the ability to scavenge oxygen free radicals, reduce the oxygen free radical stimulation and damage to the myocardium, reduce the levels of AGEs and myocardial fibrosis, and delay the process of diabetic myocardial fibrosis (Li, 2011). *G. lucidum* polysaccharides combined with metformin prevent myocardial fibrosis efficiently by strengthening antioxidant enzyme activities and downregulating myocardial CTGF, and the effect of this combination is better than that of the individual drugs at the same dose (Qiao et al., 2014).

The refractory wounds of DM patients constitute severe complications that usually result in amputation with restricted therapeutic schedules. Polysaccharide from *G. lucidum* improves wound angiogenesis and improves delayed wound healing in STZ-induced T1DM mice by partly suppressing cutaneous MnSOD nitration, p66Shc and OS in mitochondria (Tie et al., 2012). Local application of *Phallus impudicus* mycelium polysaccharides to full-thickness cutaneous wounds can enhance healing with regards to the epithelialization, contraction and growth of granulation tissue (Vyacheslav et al., 2019).

Osteopenia and osteoporosis are the two most prevalent skeletal disorders in DM patients, and DM has been shown to be an independent risk factor for fracture (Leslie et al., 2012). *Trametes versicolor* polysaccharides alleviate bone deterioration induced by DM by reducing loss of femoral cortical porosity and increasing femoral bone strength, trabecular number and bone volume of the proximal tibia in diabetic rats. The protective effects of *Trametes versicolor* polysaccharides on bone properties are partly mediated by improving hyperglycemic control (Chen et al., 2015).

## Research on the Application and Limitations of Mushroom Polysaccharides

In this section, we aimed to discuss the interesting and potential applications of mushroom polysaccharides. Currently, mushrooms are consumed as medicines (called mushroom pharmaceuticals) or as foods (in the form of dietary supplements) (Reis et al., 2017). The edibility of compounds obtained from fungi is one of their advantages; to some extent, they are safe regarding toxic effects. Fungi and medicinal mushrooms can produce 126 medicinal functions, including antidiabetic, antihypercholesterolemia, antitumor, cardiovascular, immunomodulatory, radical scavenging, antioxidant, and hepatoprotective effects. Biologically active polysaccharides exist in fruit the bodies/cultured broth/cultured mycelium of numerous higher Basidiomycetes mushrooms. Phase I, II, and III clinical trials of several mushroom polysaccharide compounds that are successfully used as drugs for diseases in Asia have been conducted (Chang and Wasser, 2012). *Agaricus brasiliensis* Ka21, a type of higher Basidiomycete, is deemed to be a safe immunostimulant mediator of biochemical parameters relevant to diabetes and obesity. Safety studies of *A. brasiliensis* Ka21 have been carried out to confirm its safety as a functional food (Yamanaka et al., 2013).

The enormous potential of mushroom polysaccharides has been discussed in this review; however, there are still some limitations to the use and consumption of mushroom polysaccharides. (1) Currently, there are few studies on mushroom polysaccharides in the treatment of diabetes complications. Most of these limited studies on mushroom polysaccharides have focused on lipid metabolism and diabetic renal complications. A small number of studies have focused on myocardial fibrosis, wound angiogenesis and bone loss; however, almost no studies have concentrated on other complications, such as diabetic foot, retinopathy and complications of the nervous system. (2) The priority of clinical studies on mushroom polysaccharides as drugs for diabetic complications needs to be showing their efficacy and safety. (3) At present, the reported species of mushrooms with pharmaceutical activities are limited, and new effective mushrooms need to be found. (4) Almost all available reports focused on the study of crude polysaccharides or polysaccharide-enriched fractions, and few studies have investigated the structure-function relationships of currently available mushroom polysaccharides. Further exploration of the relevance between the structure of mushroom polysaccharides and their therapeutic effects on diabetic complications is warranted. (5) Mushroom resources are being exploited and utilized by pharmaceutical, food industry, agricultural and other industries. We need to pay more attention to protecting intellectual property because of the potential financial and monetary values.

## Conclusions

A growing body of research suggests that mushroom polysaccharides play a key role in the treatment of diabetic complications. Using mushroom polysaccharides as agents for diabetes and its complications has various advantages. Bioformulations and herbals are considered treatment choices for multiple diseases in the new age of drug development. Further investigations and studies on the safety aspects of mushroom polysaccharides as products for diabetic complications are necessary.

## Author Contributions

Conceptualization: DW. Methodology, validation, formal analysis, and investigation: XJ and WM. Writing—original draft preparation: XJ, LL, and DW. Writing—review and editing: XJ and DW. Supervision: ZM. Funding acquisition: DW.

## Funding 

This research was funded by the Key Project on R&D of Ministry of Science and Technology (no. 2018YFE0107800) and the International Cooperation Project of Jilin Province (no. 20180414036GH).

## Conflict of Interest

The authors declare that the research was conducted in the absence of any commercial or financial relationships that could be construed as a potential conflict of interest.

## Abbreviations

CoA, Acetyl-coenzyme A; ATP, Adenosine triphosphate; AGEs, Advanced glycation end products; SVD, Cerebral small vessel disease; DM, Diabetes mellitus; DRFUs, Diabetes-related foot ulcers; DD, Diabetic dyslipidemia; DF, Diabetic foot; DKA, Diabetic ketoacidosis; DME, Diabetic macular edema; DN, Diabetic nephropathy; DPN, Diabetic peripheral neuropathy; DR, Diabetic retinopathy; FCPD, Fibrocalculous pancreatic diabetes mellitus; FAA, Free fatty acid; GDM, Gestational diabetes mellitus; HDL-C, High-density lipoprotein cholesterol; HHS, Hyperosmolar hyperglycemic status; IR, Insulin resistance; LDL-C, Low-density lipoprotein cholesterol; MDA, Malondialdehyde; MODY, Maturity-onset diabetes of the young; OS, Oxidative Stress; PKC, Protein kinase C; ROS, Reactive oxygen species; RAS, Renin-angiotensin system; SS, Silent stroke; STZ, Streptozotocin; TGs, Triglycerides; T1DM, Type 1 diabetes mellitus; T2DM, Type 2 diabetes mellitus.
