# Supplementary material for: Adjuvant Therapy With Mushroom Polysaccharides for Diabetic Complications
Source: Front Pharmacol. 2020 Feb 28;11:168. doi: 10.3389/fphar.2020.00168 (PMC7059129; doi:10.3389/fphar.2020.00168)
Supplement: Supplementary file 1 [file Table_1.docx]

**Table S1.** Effects and side effects of medications for diabetes.

| Type | Drug | Effects | Adverse effects | References |
| --- | --- | --- | --- | --- |
| Sulfonylureas | Glibenclamide | Increases plasma insulin levels and stimulates glucose metabolism. | Hypoglycemia | (1) |
|  | Glimepiride | Regulates pancreatic ATP-sensitive potassium (KATP) channels and then promote insulin release. | Hypoglycemia | (2, 3) |
| α-Glucosidase inhibitors | Miglitol | Delays the absorption of complex carbohydrates, thereby inhibiting postprandial blood glucose peaks. | Flatulence, diarrhea and stomach pain | (4) |
|  | Acarbose | Increases reversion of impaired glucose tolerance to normal glucose tolerance. | Flatulence, diarrhea | (5) |
|  | Voglibose | Inhibits the activity of human intestinalα-glucosidase enzymes, preventing the postmeal spike in plasma glucose and insulin secretion. | Most involve the lumen of the gastrointestinal tract, malabsorption | (6) |
| Thiazolidinediones | Troglitazone | Makes muscle, liver and adipose tissue sensitive to insulin and reduces insulin resistance. | Hepatotoxicity, myocardial infarct and heart failure | (7, 8) |
|  | Rosiglitazone | Reduces fasting and postprandial blood glucose levels and reduces glycated hemoglobin. | Weight gain, edema and heart failure | (9, 10) |
|  | Pioglitazone | Induces insulin sensitization, can increase insulin-dependent glucose treatment, and reduces liver glucose production. | Heart failure | (11) |
| Meglitinide analogues | Repaglinide | Stimulates insulin release through the closure of ATP-sensitive potassium channels in β-cells. | Mild hypoglycemia and weight gain | (12) |
|  | Nateglinide | Increases insulin response to postprandial blood glucose, reducing glycated hemoglobin and fasting blood glucose (FPG) levels. | Mild hypoglycemia, weight gain, upper respiratory tract infection, joint pain, sinusitis, constipation, headache and vomiting | (13) |
| Biguanides | Metformin | Decreases hepatic glucose output and reduces fasting glycemia. | Gastrointestinal side effects | (14) |
|  | Phenformin | Inhibits glucose and amino acid transport across the small bowel, enhances glycolysis in extrahepatic tissues, and inhibits hepatic gluconeogenesis. | Lactic acidosis | (15) |
| New drugs with new targets | DPP-4 inhibitors | Inhibits DPP-4 activity and increases the postprandial active incretin (GLP-1, GIP) concentration, increases insulin secretion (glucose dependence) and reduces glucagon secretion (glucose dependence). | Urticaria and angioedema | (16) |
|  | GLP-1 analogues | Has potent glucose-dependent insulinotropic and glucagonostatic actions, lowers plasma glucose levels and reduces glycemic excursions. | Loss of appetite and weight loss | (17) |
|  | GPR 119 agonists | Enhances the stimulation of insulin secretion in a glucose-dependent manner. | Reduced food intake | (18, 19) |
|  | SGLT-2 inhibitors | Reduces blood glucose by inducing glycosuria. | Genitourinary infections,  diabetic ketoacidosis | (20) |

References

1. Chang JH, Tseng CF, Wang JY. Hypoglycemia-induced myocardial infarction: an unusual adverse effect of sulfonylureas. *Int J Cardiol* (2007) 115(3):414-6. Epub 2006/06/22. doi: 10.1016/j.ijcard.2006.01.062. PubMed PMID: 16787671.

2. Holstein A, Plaschke A, Egberts EH. Lower incidence of severe hypoglycaemia in patients with type 2 diabetes treated with glimepiride versus glibenclamide. *Diabetes Metab Res Rev* (2001) 17(6):467-73. Epub 2002/01/05. PubMed PMID: 11757083.

3. Deacon CF, Lebovitz HE. Comparative review of dipeptidyl peptidase-4 inhibitors and sulphonylureas. *Diabetes Obes Metab* (2016) 18(4):333-47. PubMed PMID: WOS:000372509800002.

4. Van de Laar FA, Lucassen PLBJ, Akkermans RP, Van de Lisdonk EH, Rutten GEHM, Van Weel C. Alpha-glucosidase inhibitors for type 2 diabetes mellitus (Review) - art. no. CD003639.pub2. *Cochrane Db Syst Rev* (2005) (2). PubMed PMID: WOS:000232199200031.

5. Chiasson JL, Josse RG, Gomis R, Hanefeld M, Karasik A, Laakso M, et al. Acarbose for prevention of type 2 diabetes mellitus: the STOPNIDDM randomised trial. *Lancet* (2002) 359(9323):2072-7. doi: 10.1016/s0140-6736(02)08905-5. PubMed PMID: WOS:000176194600008.

6. Kaku K. Efficacy of voglibose in type 2 diabetes. *Expert Opin Pharmacother* (2014) 15(8):1181-90. doi: 10.1517/14656566.2014.918956. PubMed PMID: WOS:000336450900011.

7. Kahn SE. Glycemic durability of rosiglitazone, metformin, or glyburide monotherapy (vol 355, pg 2427, 2006). *N Engl J Med* (2007) 356(13):1387-8. PubMed PMID: WOS:000245221000039.

8. Khan MA, St Peter JV, Neafus KL, Hall KM, Madden MA, Duntley J, et al. A prospective, randomized comparison of the metabolic effects of pioglitazone vs rosiglitazone in patients with type 2 diabetes who were previously treated with troglitazone. *Diabetes* (2001) 50:A119-A. PubMed PMID: WOS:000168964300481.

9. Leahy JL. Glycemic Durability of Rosiglitazone, Metformin, or Glyburide Monotherapy. *Yearbook of Endocrinology* (2007) 2007:60-3. doi: 10.1016/s0084-3741(08)70035-0.

10. Sharma SK, Verma SH. A Study of Effects of Pioglitazone and Rosiglitazone on Various Parameters in Patients of Type-2 Diabetes Mellitus with Special Reference to Lipid Profile. *The Journal of the Association of Physicians of India* (2016) 64(9):24-8. PubMed PMID: MEDLINE:27762511.

11. Della-Morte D, Palmirotta R, Rehni AK, Pastore D, Capuani B, Pacifici F, et al. Pharmacogenomics and pharmacogenetics of thiazolidinediones: role in diabetes and cardiovascular risk factors. *Pharmacogenomics* (2014) 15(16):2063-82. doi: 10.2217/pgs.14.162. PubMed PMID: WOS:000346486500010.

12. Damsbo P, Clauson P, Marbury TC, Windfeld K. A double-blind randomized comparison of meal-related glycemic control by repaglinide and glyburide in well-controlled type 2 diabetic patients. *Diabetes care* (1999) 22(5):789-94. doi: 10.2337/diacare.22.5.789. PubMed PMID: WOS:000080102900023.

13. Rosenstock J, Hassman DR, Madder RD, Brazinsky SA, Farrell J, Khutoryansky N, et al. Repaglinide versus nateglinide monotherapy - A randomized multicenter study. *Diabetes care* (2004) 27(6):1265-70. doi: 10.2337/diacare.27.6.1265. PubMed PMID: WOS:000221690700002.

14. Nathan DM, Buse JB, Davidson MB, Ferrannini E, Holman RR, Sherwin R, et al. Medical Management of Hyperglycemia in Type 2 Diabetes: A Consensus Algorithm for the Initiation and Adjustment of Therapy A consensus statement of the American Diabetes Association and the European Association for the Study of Diabetes. *Diabetes care* (2009) 32(1):193-203. doi: 10.2337/dc08-9025. PubMed PMID: WOS:000262188000042.

15. Krall LP, Chabot VA. Oral Hypoglycemic Agent Update. *Medical Clinics of North America* (1978) 62(4):681-94. doi: 10.1016/s0025-7125(16)31765-5.

16. Inzucchi SE, Bergenstal RM, Buse JB, Diamant M, Ferrannini E, Nauck M, et al. Management of hyperglycaemia in type 2 diabetes: a patient-centered approach. Position statement of the American Diabetes Association (ADA) and the European Association for the Study of Diabetes (EASD). *Diabetologia* (2012) 55(6):1577-96. doi: 10.1007/s00125-012-2534-0. PubMed PMID: WOS:000303808900005.

17. Deacon CF. Therapeutic strategies based on glucagon-like peptide 1. *Diabetes* (2004) 53(9):2181-9. doi: 10.2337/diabetes.53.9.2181. PubMed PMID: WOS:000223681300001.

18. Sakairi M, Kogami M, Torii M, Kuno Y, Ohsawa Y, Makino M, et al. Synthesis and biological evaluation of a 6-aminofuro[3,2-c]pyridin-3(2H)-one series of GPR 119 agonists. *Arzneimittelforschung* (2012) 62(11):537-44. Epub 2012/09/14. doi: 10.1055/s-0032-1323760. PubMed PMID: 22972470.

19. Jones RM, Leonard JN, Buzard DJ, Lehmann J. GPR119 agonists for the treatment of type 2 diabetes. *Expert Opin Ther Patents* (2009) 19(10):1339-59. doi: 10.1517/13543770903153878. PubMed PMID: WOS:000271085300001.

20. Santos LL, Lima FJC, Sousa-Rodrigues CF, Barbosa FT. Use of SGLT-2 inhibitors in the treatment of type 2 diabetes mellitus. *Rev Assoc Med Bras (1992)* (2017) 63(7):636-41. Epub 2017/10/05. doi: 10.1590/1806-9282.63.07.636. PubMed PMID: 28977090.
